# Social Health Is Associated With Tract-Specific Brain White Matter Microstructure in Community-Dwelling Older Adults

**DOI:** 10.1016/j.bpsgos.2022.08.009

**Published:** 2022-09-08

**Authors:** Andrea Costanzo, Isabelle F. van der Velpen, M. Arfan Ikram, Myrra J.F. Vernooij-Dassen, Wiro J. Niessen, Meike W. Vernooij, Martien J. Kas

**Affiliations:** a Groningen Institute for Evolutionary Life Sciences, Faculty of Science and Engineering, University of Groningen, Groningen, the Netherlands; b Department of Epidemiology, Erasmus MC University Medical Center, Rotterdam, the Netherlands; c Department of Radiology and Nuclear Medicine, Erasmus MC University Medical Center, Rotterdam, the Netherlands; d Department of IQ Healthcare, Radboud University Medical Center, Nijmegen, the Netherlands

**Keywords:** Diffusion tensor imaging, Loneliness, Social health, Social support, White matter microstructure, White matter tracts

## Abstract

**Background:**

Poor social health has been linked to a risk of neuropsychiatric disorders. Neuroimaging studies have shown associations between social health and global white matter microstructural integrity. We aimed to identify which white matter tracts are involved in these associations.

**Methods:**

Social health markers (loneliness, perceived social support, and partnership status) and white matter microstructural integrity of 15 white matter tracts (identified with probabilistic tractography after diffusion magnetic resonance imaging) were collected for 3352 participants (mean age 58.4 years, 54.9% female) from 2002 to 2008 in the Rotterdam Study. Cross-sectional associations were studied using multivariable linear regression.

**Results:**

Loneliness was associated with higher mean diffusivity (MD) in the superior thalamic radiation and the parahippocampal part of the cingulum (standardized mean difference for both tracts: 0.21, 95% CI, 0.09 to 0.34). Better perceived social support was associated with lower MD in the forceps minor (standardized mean difference per point increase in social support: −0.06, 95% CI, −0.09 to −0.03), inferior fronto-occipital fasciculus, and uncinate fasciculus. In male participants, better perceived social support was associated with lower MD in the forceps minor, and not having a partner was associated with lower fractional anisotropy in the forceps minor. Loneliness was associated with higher MD in the superior thalamic radiation in female participants only.

**Conclusions:**

Social health was associated with tract-specific white matter microstructure. Loneliness was associated with lower integrity of limbic and sensorimotor tracts, whereas better perceived social support was associated with higher integrity of association and commissural tracts, indicating that social health domains involve distinct neural pathways of the brain.

Social health, which entails how the interaction between the direct social environment and the individual affects the individual’s perception of social life as a domain of general well-being (1, 2, 3, 4), is fundamental for human survival and heavily dependent on complex neurocognitive systems (5). Accordingly, social dysfunction is one of the first and most common signs of major neuropsychiatric disorders (6). Importantly, poor social health is also associated with higher mortality and morbidity risk, including risk of dementia (7, 8, 9, 10, 11, 12, 13).

To understand the complex relations between social health and the brain in the risk of neuropsychiatric disorders, numerous neuroimaging studies have been performed, focusing recently especially on white matter (14, 15, 16). A recent study showed that social health is associated with global white matter microstructure in a general population of older adults (17). Nevertheless, the relationship between social health and specific white matter tracts remains unclear.

Research in clinical populations of patients with conditions characterized by symptoms in the social domain have shown that schizophrenia (18, 19, 20), autism spectrum disorder (14,21), social anxiety (22), and social cognition deficits (23) are associated with poor microstructure of specific white matter tracts such as the frontal and temporal thalamic projections (14), corpus callosum (23,24), uncinate fasciculus (14,19, 20, 21,23), superior and inferior longitudinal fasciculus (22,23), and inferior fronto-occipital fasciculus (19). In addition, some studies have focused on particular aspects of the social life of individuals, such as social network size (25) and diversity (26), indicating that there may be differentiated relationships between different social domain components and specific neural circuits and that these relationships may show sex differences (27).

Based on these findings, social health may be associated with the integrity of specific white matter tracts in the general population. Knowledge of this association is instrumental to better understand the neurobiological mechanisms that underlie neuropsychiatric disorders such as dementia and schizophrenia (6). More specifically, suboptimal social health may contribute to the risk of neuropsychiatric disorders through changes in specific white matter tracts.

Therefore, the aim of this study was to identify associations between different aspects of social health and tract-specific white matter microstructure in community-dwelling older adults. We hypothesized that worse social health would be associated with worse integrity of white matter microstructure in tracts that are important for cognitive functioning.

## Methods and Materials

### Design and Population

This study was embedded in the Rotterdam Study, a prospective, population-based cohort study based in Rotterdam, the Netherlands. It started in 1990 and is ongoing (28). Persons 40 years of age or older living in the Ommoord neighborhood were invited to participate and were followed every 3 to 4 years. The Rotterdam Study has been approved by the Medical Ethics Committee of the Erasmus MC (registration number MEC 02.1015) and by the Dutch Ministry of Health, Welfare and Sport (Population Screening Act WBO, license number 1071272-159521-PG). The Rotterdam Study Personal Registration Data collection is filed with the Erasmus MC Data Protection Officer under registration number EMC1712001. The Rotterdam Study has been entered into the Netherlands National Trial Register (NTR) (http://www.trialregister.nl) and the World Health Organization’s International Clinical Trials Registry Platform (https://apps.who.int/trialsearch/) under shared catalog number NL6645/NTR6831. All participants provided written informed consent to participate in the study and to have their information obtained from treating physicians.

Social health markers were collected from January 2002 to November 2008. Magnetic resonance imaging (MRI) data were collected from March 2006 to March 2010. The median time difference between assessment of social health markers and MRI scan was 90 days (interquartile range 57–195 days). Diffusion tensor imaging (DTI) processing was complete for 3526 participants, of whom 3488 participants had complete information on social health markers. After exclusion of participants with prevalent dementia (*n* = 53) and cortical brain infarcts (*n* = 83), 3352 participants were included in the final study sample.

### Social Health Markers

Social health markers collected during the home interview in the Rotterdam Study are loneliness, perceived social support, and partnership status. Loneliness and perceived social support are subjective measures of the perception of social life, while partnership status is an objective measure that can influence an individual’s perception of social life and thus social health. Loneliness, defined as “the subjective experience of an unpleasant lack of (quality of) social relationships,” was assessed with a single-item question from the Center for Epidemiological Studies Depression scale (CES-D) (29). We dichotomized the responses into lonely (feelings of loneliness ≥ 1 day/week) and not lonely (feelings of loneliness < 1 day/week). Perceived social support was assessed with a 5-item questionnaire modified from the Health and Lifestyle Survey. The questions were “I know people, among my family and friends, 1) who do things that make me happy; 2) whom I can always count on; 3) who would make sure that I would get help if I would need it; 4) who give me the feeling that I am important in their lives; and 5) who accept me for who I am.” Participants had the following options for each question: agree, somewhat agree, or disagree. The sum scores ranged from 0 to 10, with a higher value corresponding to better perceived social support. Scores were weighted to account for responses with 1 missing item. Scores with fewer than 4 responses were excluded. Cronbach’s alpha for the social support questionnaire was 0.74 (95% CI, 0.73 to 0.74). The answer options for the partnership status question were “married/has a partner,” “widowed/divorced,” and “never married,” which were subsequently dichotomized into “has a current partner” and “does not have a current partner.”

### MRI Acquisition and Processing

Brain MRI was performed for all participants on a single 1.5T MRI scanner (GE Signa Excite) with an 8-channel head coil and without any major hardware or software upgrades during the time of the study. A detailed protocol of MRI in the Rotterdam Study including quality control has been extensively described previously (30). In brief, structural imaging included a T1-weighted sequence, a proton density–weighted sequence, a T2-weighted fluid-attenuated inversion recovery sequence, and a 3-dimensional T2∗-weighted gradient-recalled echo scan. Diffusion-weighted imaging is described below. Due to a technical issue, a subset of 1312 participants was scanned with phase and frequency encoding directions switched for the DTI sequence between February 2007 and May 2008. This introduced a mild ghosting artifact in the phase encoding direction, which was adjusted for in statistical analysis (see Statistical Analysis). Brain volumetric measures (gray matter, white matter, and cerebrospinal fluid) were quantified using a k-nearest neighbor algorithm (31). White matter hyperintensities were quantified with an automated postprocessing step based on the tissue segmentation and fluid-attenuated inversion recovery image (32). All tissue segmentations were inspected for quality by trained raters and manually corrected when necessary. Presence of cortical brain infarcts was visually assessed by trained raters.

### Diffusion-MRI Processing and Tractography

**Figure 1:**
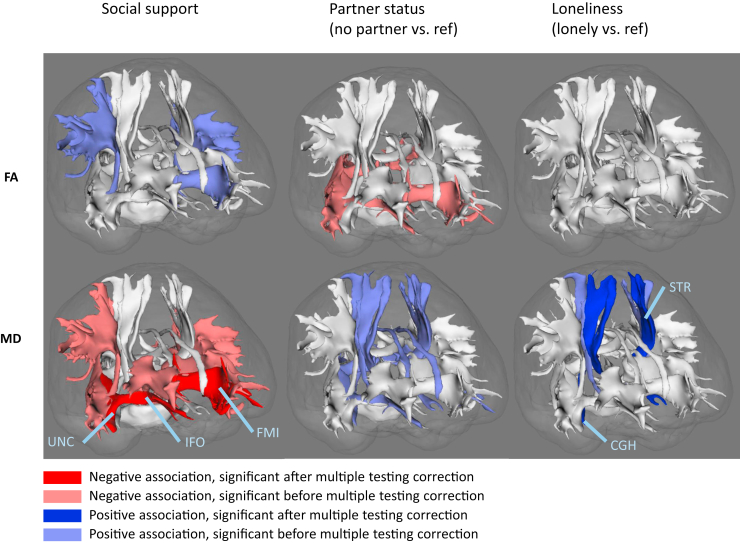
Tract specific white matter microstructure and social health. Standardized mean differences of model 3, for fractional anisotropy (FA) (top row) and mean diffusivity (MD) (bottom row), for all 15 white matter tracts presented in a single-subject anatomy for visualization. Significant associations after multiple testing correction (*p* < .0039, Šidák correction) in darker color, significant associations before multiple testing correction (*p* < .05) in lighter color; see key. Nonsignificant associations are displayed in white. CGH, parahippocampal part of cingulum; FMI, forceps minor; IFO, inferior fronto-occipital fasciculus; ref, reference; STR, superior thalamic radiation; UNC, uncinate fasciculus.

To obtain DTI data, we performed a single shot, diffusion-weighted spin-echo echo-planar imaging sequence. The maximum b value was 1000 s/mm^2^ in 25 noncollinear directions; 3 volumes were acquired without diffusion weighting (b = 0 s/mm^2^). All diffusion data were preprocessed using a standardized pipeline (33). In short, eddy current and head motion corrections were performed on the diffusion-weighted volumes. Diffusion tensors were estimated using a nonlinear Levenberg Marquardt estimator available in ExploreDTI (34). Tensors were fit using the resampled data, which allowed computation of global mean fractional anisotropy (FA) and mean diffusivity (MD) in the normal-appearing white matter in combination with the tissue segmentation. Next, diffusion data were used to segment white matter tracts using a probabilistic tractography approach, which has been described in detail previously (35). In brief, tractography was performed using PROBTRACKX (36), which is a probabilistic Bayesian framework for white matter tractography and is available in FSL (version 4.1.4) (37). For 15 different white matter tracts (12 of which were segmented bilaterally), tract-specific white matter microstructural diffusion-MRI parameters (median FA and MD) were obtained with subsequent averaging of left and right measures. Average reproducibility (*R*^2^) of tract-specific measurements was 0.87 (35). Tracts were categorized, based on anatomy or presumed function, into association tracts (anterior thalamic radiation, inferior fronto-occipital fasciculus, inferior longitudinal fasciculus, posterior thalamic radiation, superior longitudinal fasciculus, and uncinate fasciculus), commissural tracts (forceps major and forceps minor), limbic system tracts (cingulate gyrus part of the cingulum, parahippocampal part of cingulum, and fornix), and sensorimotor tracts (corticospinal tract, middle cerebellar peduncle, medial lemniscus, and superior thalamic radiation) (Figure 1). Tract segmentations were used to obtain tract-specific white matter volumes and tract-specific white matter hyperintensity volumes by combining the tissue and tract segmentation. The cerebellum could not be fully incorporated in the field of view of the diffusion-MRI scan, resulting in partial coverage of the medial lemniscus at the lower border of the scan. Alternative seed masks for tractography were selected until reasonable coverage was achieved to overcome this problem (35). This correction was treated as a potential confounder in all models that included the medial lemniscus. Further information on quality control for DTI and white matter tract segmentation can be found in Supplemental Methods.

A better microstructure is characterized by high FA and low MD (38). FA values were computed based on the ratio between the longest and shortest axes of diffusion, giving values between 0 and 1, with 0 indicating isotropic diffusion (equal in all directions) and an absence of organized fiber tracts to constrain directionality. A value closer to 1 means that diffusion occurs more strongly along one direction, suggesting increased fiber organization and white matter integrity.

### Other Measurements

We selected covariates in our study based on their potential to be a cause of the exposure or the outcome, or both, or as a proxy of unmeasured confounding. Intracranial volume was defined as the sum of the total brain volume and cerebrospinal fluid on MRI. Educational attainment was assessed during the baseline interview and categorized according to United Nations Educational, Scientific and Cultural Organization classification criteria. Cognitive function was assessed at the research center using the Mini-Mental State Examination. Smoking status was assessed during the interview, and study participants were categorized as former, never, or current smokers. Alcohol consumption was classified into none (no alcoholic beverages), moderate (≤1 beverage/day), and heavy (>1 beverage/day) based on grams of alcohol consumed per day calculated from interview data on number and type of alcoholic beverages consumed. Body mass index (kg/m^2^) was calculated from body height and weight assessed at the research center.

Hypertension was defined as a systolic blood pressure >140 mmHg or a diastolic blood pressure >90 mmHg or taking antihypertensive medication. Blood pressure was measured twice in sitting position using a random-zero sphygmomanometer at the research center. The average of the two measurements was used to define hypertension. Diabetes mellitus was determined based on fasting serum glucose level (>7.0 mmol/L) or, if that was unavailable, on nonfasting serum glucose level (>11.1 mmol/L), or the use of antidiabetic medication. The ascertainment of coronary heart disease, heart failure, and clinical stroke diagnoses was based on medical records and has been described in detail elsewhere (28).

Depressive symptoms were assessed during the home interview using the CES-D. CES-D scores were weighted to account for missing responses if missing values were <25% (29,39). The presence of anxiety disorders was assessed using an adapted version of the Munich Composite International Diagnostic Interview to obtain diagnoses of generalized anxiety disorder, agoraphobia, social phobia, panic disorder, and specific phobias in accordance with DSM-IV (40).

### Statistical Analysis

Missing covariate data were imputed with 5-fold multiple imputation. FA and MD were standardized for each tract to facilitate comparison of associations. We used the mean FA and MD values of the left and right hemispheres combined to limit the number of outcomes and the risk of spurious findings. We used multivariable linear regression models to study cross-sectional associations between social health markers and tract-specific FA and MD. We performed stepwise adjustment of the models to interpret the change of the effect estimates with each addition of a set of covariates.

In model 1, we adjusted for age, sex, and intracranial volume. We included phase encoding direction of the diffusion scan as a covariate and potential confounder in all models. In model 2, we added smoking status, alcohol consumption, body mass index, hypertension, education level, Mini-Mental State Examination, CES-D score, presence of anxiety disorders, coronary heart disease, diabetes diagnosis, heart failure diagnosis, and clinical stroke diagnosis. In model 3, we additionally adjusted the model for tract volume and tract-specific white matter hyperintensity volume. Next, we stratified all models on sex to assess effect modification by sex by interpreting the associations separately for male and female participants. Additive interaction was studied by adding an interaction term for the product of each social health marker with sex to each model.

We performed permutation testing to determine the number of independent tests, which is important considering that we included 3 determinants and 15 outcomes that are theoretically correlated. For each outcome variable, linear regressions were run with a random variable and repeated 10,000 times. The minimum *p* value for each regression model (permutation) was extracted, and these *p* values were sorted to define the significance threshold, which was based on the 5% quantile (.0037). We then divided .05 by this threshold to obtain the number of independent tests (*n* = 13). We calculated the new significance threshold using Šidák correction, α_*n*_ = 1 − (1 − α)^(1/^^*n*^^)^, where *n* is the number of independent tests, resulting in a multiple-testing adjusted *p-*value threshold of .0039 (41).

To investigate whether the social health markers are independent determinants by mutually adjusting them, we performed a sensitivity analysis including all social health markers in the same linear regression model. Correlations between social health markers are presented in Figure S1.

## Results

The characteristics of the study sample (*N* = 3352) are shown in Table 1. Participants’ mean age was 58.4 years (SD 11.7), and 54.9% of the participants were female. Eighty-one percent of the participants had a partner, 88% reported not feeling lonely, and the median social support score was 10 (interquartile range 10–10, variance 0.82).

**Table 1:** Characteristics of the Study Sample

Characteristic	Overall, *N* = 3352
Age, Years, Mean (SD)	58.4 (7.1)
Sex, Female, *n* (%)	1841 (54.9%)
Loneliness, Lonely, *n* (%)	396 (11.8%)
Perceived Social Support, Weighted Score, Median (IQR)	10 (10–10)
Perceived Social Support, Weighted Score, Mean (SD)	9.7 (0.9)
Marital Status, *n* (%)	
Married or has partner	2711 (80.9%)
No current partner	641 (19.1%)
Education, *n* (%)	
Primary education	273 (8.1%)
Lower/intermediate general education or lower vocational education	1211 (36.1%)
Intermediate vocational education or higher general education	987 (29.4%)
Higher vocational education or university	881 (26.3%)
MMSE Score, Median (IQR)	29.0 (27.0–29.0)
Depressive Symptoms, CES-D Score, Median (IQR)	3.0 (1.0–7.0)
Clinically Relevant Depressive Symptoms, CES-D ≥ 16, *n* (%)	281 (8.4%)
Anxiety Disorder, Present, *n* (%)	255 (7.6%)
Smoking Status, *n* (%)	
Never	1014 (30.3%)
Former	1542 (46.0%)
Current	796 (23.7%)
Alcohol Use, *n* (%)	
None	334 (10.0%)
Moderate, 0–1 units per day	2029 (60.5%)
Heavy, >1 unit per day	989 (29.5%)
Body Mass Index, kg/m^2^, Mean (SD)	27.5 (4.1)
Hypertension, *n* (%)	1765 (52.3%)
History of Diabetes Mellitus Type 2, *n* (%)	329 (9.8%)
History of Coronary Heart Disease, *n* (%)	120 (3.6%)
History of Heart Failure, *n* (%)	24 (0.7%)
History of Clinical Stroke, *n* (%)	48 (1.4%)

CES-D, Center for Epidemiological Studies Depression scale; IQR, interquartile range; MMSE, Mini-Mental State Examination.

Associations between social health markers and tract-specific white matter integrity are presented in Figure 1 and Table 2. After adjusting for all covariates, loneliness was associated with a higher MD of the parahippocampal part of the cingulum (standardized mean difference: 0.21, 95% CI, 0.09 to 0.34) and the superior thalamic radiation (standardized mean difference: 0.21, 95% CI, 0.09 to 0.34). Associations between loneliness and tract-specific FA were not statistically significant after multiple testing correction.

**Table 2:** Associations Between Social Health Markers and Tract-Specific White Matter Integrity

	Model	Association Tracts						Commissural Tracts		Limbic Tracts			Sensorimotor Tracts			
Social Health Markers		ATR	IFO	ILF	PTR	SLF	UNC	FMA	FMI	CGC	CGH	FX	CST	MCP	ML	STR
Fractional Anisotropy																
Social support, per point increase	1	0.01	0.04^a^	0.04^a^	0.05^a^	0.04^a^	0.04^a^	0.04^a^	0.05^b^	0.01	0.01	0.01	0.02	−0.02	0.02	0.03
Social support, per point increase	2	0.01	0.03	0.04	0.04^a^	0.04^a^	0.04^a^	0.03	0.05^a^	0.01	0	0	0.01	−0.02	0.01	0.03
Social support, per point increase	3	0	0.03	0.03	0.02	0.03^a^	0.02	0.01	0.04^a^	0	0	0.01	0	−0.02	0.02	0.02
Partner status, no partner vs. partner	1	−0.06	−0.14^b^	−0.13^b^	0.16^b^	−0.08	−0.06	−0.13^b^	−0.12^b^	−0.06	−0.04	0.05	−0.03	0.02	−0.03	−0.05
Partner status, no partner vs. partner	2	−0.04	−0.12^a^	−0.11^a^	−0.1 b	−0.06	−0.05	−0.1^a^	−0.1^a^	−0.05	−0.03	0.06	−0.01	0.01	−0.02	−0.02
Partner status, no partner vs. partner	3	0	−0.09^a^	−0.08^a^	−0.1^a^	−0.03	−0.02	−0.03	−0.08^a^	−0.06	−0.04	0.08^a^	−0.01	0.01	−0.01	−0.01
Loneliness, lonely vs. not lonely	1	−0.11^a^	−0.12^a^	−0.1^a^	−0.14^a^	−0.07	−0.09	−0.1^a^	−0.08	−0.1	−0.06	−0.07	−0.08	0.06	−0.03	−0.03
Loneliness, lonely vs. not lonely	2	−0.1	−0.1	−0.07	−0.09	−0.07	−0.13^a^	−0.05	−0.05	−0.1	−0.06	−0.06	−0.04	0.02	−0.00	0.02
Loneliness, lonely vs. not lonely	3	−0.07	−0.07	−0.06	−0.08	−0.03	−0.07	0	−0.02	−0.08	−0.07	−0.04	−0.01	0	0.01	0.04
Mean Diffusivity																
Social support, per point increase	1	−0.04^a^	−0.06^b^	−0.05^a^	−0.04^a^	−0.05^a^	−0.06^b^	−0.03	−0.07^b^	−0.04^a^	−0.02	−0.05^b^	−0.04^a^	0.01	−0.05^a^	−0.02
Social support, per point increase	2	−0.03^a^	−0.06^b^	−0.05^a^	−0.04^a^	−0.04^a^	−0.06^b^	−0.03	−0.06^b^	−0.03	−0.01	−0.04^a^	−0.04^a^	0.01	−0.04^a^	−0.02
Social support, per point increase	3	−0.02^a^	−0.05^b^	−0.04^a^	−0.02	−0.04^a^	−0.05^b^	−0.01	−0.06^b^	−0.03	−0.01	−0.02	−0.03	0.01	−0.04^a^	−0.01
Partner status, no partner vs. partner	1	0.11^b^	0.11^b^	0.08 a	0.14^b^	0.11^a^	0.09 a	0.14^b^	0.08^a^	0.1^a^	0.13^b^	0.08^a^	0.13^b^	0.01	0.05	0.13^b^
Partner status, no partner vs. partner	2	0.07	0.09^a^	0.06	0.12^b^	0.08^a^	0.08	0.13^b^	0.06	0.09^a^	0.12^a^	0.06	0.11^a^	0.03	0.03	0.12^a^
Partner status, no partner vs. partner	3	0.02	0.07^a^	0.04	0.09^a^	0.06	0.06	0.07	0.04	0.09^a^	0.11^a^	−0.01	0.09^a^	0.03	0.03	0.1^a^
Loneliness, lonely vs. not lonely	1	0.08	0.13^a^	0.12^a^	0.11^a^	0.11^a^	0.07	0.11^a^	0.1^a^	0.09	0.19^b^	0.11^a^	0.17^b^	−0.03	0.03	0.16^b^
Loneliness, lonely vs. not lonely	2	0.03	0.09	0.08	0.06	0.09	0.08	0.09	0.04	0.09	0.22^b^	0.05	0.19^b^	0.03	−0.01	0.22^b^
Loneliness, lonely vs. not lonely	3	0.01	0.07	0.05	0.04	0.07	0.04	0.05	0.02	0.08	0.21^b^	−0.01	0.16^a^	0.07	−0.01	0.21^b^

Standardized mean differences of models 1, 2, and 3, for fractional anisotropy and mean diffusivity.

ATR, anterior thalamic radiation; CGC, cingulate gyrus part of cingulum; CGH, parahippocampal part of cingulum; CST, corticospinal tract; FMA, forceps major; FMI, forceps minor; FX, fornix; IFO, inferior fronto-occipital fasciculus; ILF, inferior longitudinal fasciculus; MCP, middle cerebellar peduncle; ML, medial lemniscus; PTR, posterior thalamic radiation; SLF, superior longitudinal fasciculus; STR, superior thalamic radiation; UNC, uncinate fasciculus.

a Indicates the statistically significant (*p* ≤ .05) coefficients without multiple testing correction.

b Indicates the statistically significant coefficients after multiple testing correction (Šidák correction).

Better (i.e., higher) perceived social support scores were associated with lower MD of the inferior fronto-occipital fasciculus (standardized mean difference of MD per point increase in social support: −0.05, 95% CI, −0.08 to −0.03), the uncinate fasciculus (standardized mean difference: −0.05, 95% CI, −0.08 to −0.02), and the forceps minor (standardized mean difference: −0.06, 95% CI, −0.09 to −0.03) (Table 2) (model 3).

Partnership status was no longer significantly associated with white matter tract integrity after adjusting for all covariates and multiple testing correction. Partnership status was associated with FA and MD only in model 1, in which not having a partner was associated with a lower FA for the inferior fronto-occipital fasciculus, inferior longitudinal fasciculus, posterior thalamic radiation, forceps major, and forceps minor after multiple testing correction. Not having a partner was associated with higher MD in the anterior thalamic radiation, inferior fronto-occipital fasciculus, posterior thalamic radiation, forceps major, parahippocampal part of cingulum, corticospinal tract, and superior thalamic radiation only in model 1. Not having a partner remained associated with higher MD of the posterior thalamic radiation (standardized mean difference: 0.12, 95% CI, 0.04 to 0.20) and the forceps major (standardized mean difference: 0.13, 95% CI, 0.04 to 0.21) after adjusting for lifestyle and multimorbidity, but not after adjusting for tract volume and tract-specific white matter hyperintensities volume.

After stratifying model 3 based on sex, only male participants showed an association of social support with MD of the forceps minor (standardized mean difference: −0.07, 95% CI, −0.11 to −0.03; *p* for interaction = .80) and an association of partnership status with the FA of the forceps minor (standardized mean difference: −0.22, 95% CI, −0.36 to −0.07; *p* for interaction = .02) as shown in Table 3. Female participants showed an association of loneliness with the MD of the superior thalamic radiation (standardized mean difference: 0.31, 95% CI, 0.13 to 0.50; *p* for interaction = .05).

**Table 3:** Associations Between Social Health Markers and Tract-Specific White Matter Integrity Stratified by Sex

	Sex	Association Tracts						Commissural Tracts		Limbic Tracts		Sensorimotor Tracts				
Social Health Markers		ATR	IFO	ILF	PTR	SLF	UNC	FMA	FMI	CGC	CGH	FX	CST	MCP	ML	STR
Fractional Anisotropy																
Social support, per point increase	Male	0.00	0.05	0.04	0.02	0.05^a^	0.03	0.00	0.06^a^	0.01	0.02	−0.02	0.00	−0.02	−0.01	0.03
Social support, per point increase	Female	0.00	0.00	0.01	0.03	0.02	0.01	0.02	0.03	0.01	0.02	0.01	0.02	−0.03	0.05^a^	0.00
Partner status, no partner vs. partner	Male	0.07	−0.12	−0.07	−0.07	−0.04	−0.05	−0.02	−0.22^b^^,^^c^	−0.09	−0.04	0.09	0.16^a^^,^^c^	0.04	0.12	0.11
Partner status, no partner vs. partner	Female	−0.03	−0.10^a^	−0.09	−0.10^a^	−0.03	−0.01	−0.04	−0.03	−0.05	−0.03	0.07	−0.05^c^	−0.03	−0.03	−0.06
Loneliness, lonely vs. not lonely	Male	−0.08	−0.01	0.03	−0.04	0.01	0.00	0.09	0.02	0.00	−0.06	−0.01	0.01	0.11	−0.11	0.07
Loneliness, lonely vs. not lonely	Female	−0.07	−0.09	−0.09	−0.10	−0.04	−0.09	−0.06	−0.05	−0.12	−0.07	−0.05	−0.01	−0.04	0.04	0.05
Mean Diffusivity																
Social support, per point increase	Male	−0.02	−0.05^a^	−0.03	0.00	−0.05^a^	−0.05^a^	0.01	−0.07^b^^,^^c^	−0.04	−0.02	−0.02	−0.03^a^	−0.01	−0.02	−0.02
Social support, per point increase	Female	−0.03	−0.05^a^	−0.05^a^	−0.03	−0.03	−0.05^a^	−0.02	−0.05^b^	−0.03	0.00	−0.02	−0.02	0.03	−0.07	0.00
Partner status, no partner vs. partner	Male	0.07	0.15^a^	0.02	0.07	0.07	0.12	0.16^a^	0.08	0.12	0.13	0.04	0.07	0.04	0.01	0.03
Partner status, no partner vs. partner	Female	0.02	0.05	0.03	0.09^a^	0.06	0.03	0.03	0.02	0.10	0.11^a^	−0.01	0.11	0.04	0.04	0.14^a^
Loneliness, lonely vs. not lonely	Male	−0.02	0.04	−0.07^c^	0.00	0.06	0.04	0.02	0.05	0.00	0.14	−0.01	−0.03	0.16	0.01	−0.01^c^
Loneliness, lonely vs. not lonely	Female	0.03	0.09	0.11^c^	0.08	0.06	0.04	0.07	0.00	0.12	0.22^a^	−0.01	0.24^a^	0.02	−0.02	0.31^b^^,^^c^

Standardized mean differences of model 3 stratified by sex for fractional anisotropy and mean diffusivity.

ATR, anterior thalamic radiation, CGC, cingulate gyrus part of cingulum; CGH, parahippocampal part of cingulum; CST, corticospinal tract; FMA, forceps major; FMI, forceps minor; FX, fornix; IFO, inferior fronto-occipital fasciculus; ILF, inferior longitudinal fasciculus; MCP, middle cerebellar peduncle; ML, medial lemniscus; PTR, posterior thalamic radiation; SLF, superior longitudinal fasciculus; STR, superior thalamic radiation; UNC, uncinate fasciculus.

a Indicates the statistically significant (*p* ≤ .05) coefficients without multiple testing correction.

b Indicates the statistically significant coefficients after multiple testing correction (Šidák correction).

c Indicates significant *p* values for the interaction term of the social health marker with sex.

The sensitivity analyses did not change the interpretation of our findings (Table S1). In the mutually adjusted model, the association between social support and the uncinate fasciculus was no longer statistically significant after multiple testing correction.

## Discussion

In this study, we aimed to identify associations between different aspects of social health and tract-specific brain white matter microstructure in community-dwelling older adults. We found that better perceived social support was associated with lower MD (reflecting higher structural integrity) of the inferior fronto-occipital fasciculus, uncinate fasciculus, and forceps minor, whereas loneliness was associated with higher MD (reflecting lower integrity) of the parahippocampal part of the cingulum and of the superior thalamic radiation. In addition, we found potential interaction effects of sex on the associations between partner status and loneliness with specifically the integrity of the forceps minor and superior thalamic radiation. We will briefly discuss each of these findings in this section.

Better perceived social support was associated with lower MD of the association and commissural tracts, namely the inferior fronto-occipital fasciculus, uncinate fasciculus, and forceps minor. According to two recent extensive literature review papers on structural white matter, the inferior fronto-occipital fasciculus and uncinate fasciculus may play key roles in socioemotional processing and social cognition (14,15). The inferior fronto-occipital fasciculus runs from the ventral occipital cortex through the temporal cortex and terminates in the orbitofrontal, medial prefrontal, and inferior frontal cortex. As such, it is involved in face processing and perception, mentalizing (theory of mind), and embodied cognition (mirroring) (15). The uncinate fasciculus is a limbic tract that connects medial temporal areas to the medial and lateral orbitofrontal cortex and has been linked to emotion recognition and empathic abilities (15). The forceps minor, representing the anterior commissure of the corpus callosum, connects bilateral anterior frontal regions through the genu. Malformation or disruptions of the anterior corpus callosum have been associated with deficits in cognitive functioning and social communication (42), and microstructural integrity of the forceps minor has more recently been linked to social network size and diversity (25,26). These findings indicate that the perception of social support is associated with white matter structures that are important for the adequate processing of social cues of others and for responding adequately to others’ emotions. Potentially, perceiving good social support stimulates healthy brain structures that are needed for the ability to appropriately respond to social cues.

In our study, loneliness was associated with higher MD of limbic and sensorimotor tracts, specifically the parahippocampal part of the cingulum and the superior thalamic radiation. Several previous studies have reported on associations between loneliness and white matter structure, including a recent population-based study that found that loneliness was mainly linked to impaired microstructure of the fornix, but not other structures (27). A study of young adults found that loneliness was associated with reduced white matter density in brain areas that are related to social cognition, empathy, self-cognition, and self-efficacy (43). Loneliness has not specifically been linked yet to the cingulum and thalamic radiations in previous research. The parahippocampal part of the cingulum is part of the limbic system and connects the medial prefrontal cortex and anterior cingulate cortex through the precuneus to medial temporal regions proximal to the hippocampus (15). It is involved in functions related to memory and socioemotional processing and has been suggested as part of the brain network related to empathy (15). In contrast, the superior thalamic radiation has (to our knowledge) not been mentioned before in relation to social processes. The superior thalamic radiation connects the thalamus to the parietal lobe, penetrating the posterior limb of the internal capsule, and has mainly been thought to be involved in processing sensory input. Recently, however, findings from the UK Biobank indicated that depressive symptomology and major depressive disorder are associated with altered microstructure of thalamic radiations, including the superior thalamic radiation (44). Thus, loneliness may be associated with altered processing of sensory information in the brain, with implications for brain structures required for memory and for socioemotional and sensory processing.

Partnership status was not significantly associated with tract-specific white matter integrity after adjusting for tract–white matter volume and tract–white matter hyperintensity volume, indicating that the association between partnership status and white matter integrity may be better explained by white matter macrostructure than by partnership status and covariates alone. Prior to multiple testing correction, partnership status showed an association with the microstructural integrity of several white matter tracts, including association, limbic, and sensorimotor tracts, such that not having a current partner was consistently associated with worse microstructure. Future studies with higher statistical power should investigate these associations further.

Finally, our findings suggest sex-specific differences in the associations between social health and white matter microstructure. We found that not having a partner was associated with lower FA of the forceps minor in males only and that the association between loneliness and higher MD of the superior thalamic radiation was only present in female participants. This is in line with sex differences found in previous studies, where loneliness in men has been associated with greater structural integrity of the fornix (27) and with smaller global white matter volume (17). Importantly, however, these results could be affected by the fact that men tend to report themselves lonely less often than women due to greater stigma associated with loneliness for men (45). More research is required to elucidate the complex associations between sex, gender, social health, and the brain.

The large sample size and the population-based setting are important strengths of this study. Another strength is that we controlled for a large number of potential confounders, including markers of physical and mental health, lifestyle behaviors, and tract volume.

Limitations of this study include the fact that we considered only 15 major white matter tracts, excluding minor tracts from our analysis. For tracts that are located on both right and left hemispheres, we only analyzed averaged values for the entire tracts and did not consider the portion of the tracts in the 2 hemispheres separately. We regarded FA and MD as reflecting microstructural integrity, which is a simplification of the underlying biological processes but is generally accepted as such a representation. Furthermore, the perceived social support questionnaire has not been formally validated, and the direct question on loneliness from the CES-D might have led to underreporting of loneliness in our sample. Finally, due to the cross-sectional nature of our analysis, the causal relationship between social health variables and white matter microstructure cannot be inferred by this study and should be elucidated by future investigations, which should also consider the possibility that negative (early) life experiences such as childhood maltreatment could have influenced both white matter and social health and thus might represent a possible additional mechanism underlying these findings (42,43).

### Conclusions

We investigated associations between social health markers and the microstructure of specific white matter tracts and found that better social health is related to higher microstructural integrity of specific tracts: the superior thalamic radiation, parahippocampal part of the cingulum, uncinate fasciculus, inferior fronto-occipital fasciculus, and forceps minor. Different social health markers such as loneliness and perceived social support were found to be associated with the microstructural integrity of different white matter tracts, suggesting that various social health domains influence different neural pathways of the brain. Our findings are of theoretical and practical relevance, giving new insight into the relationship between social health and white matter tracts and contributing to the understanding of the mechanisms that lead to neuropsychiatric diseases, including dementia, in which social dysfunction is a common symptom.

## Supplementary Material

Supplememtary Figure and Supplementary Table

Key Resources Table
