# Supplementary material for: Social Health Is Associated With Tract-Specific Brain White Matter Microstructure in Community-Dwelling Older Adults
Source: Biol Psychiatry Glob Open Sci. 2022 Sep 8;3(4):1003–11. doi: 10.1016/j.bpsgos.2022.08.009 (PMC10593878; doi:10.1016/j.bpsgos.2022.08.009)
Supplement: Supplememtary Figure and Supplementary Table [file mmc1.pdf]

Social Health Is Associated With Tract-Specific Brain White Matter Microstructure In Community-Dwelling Older Adults

Supplemental Information

Costanzo *et al.*

Supplemental Methods | DTI and white matter tract segmentation quality control.

Figure S1 | Correlations between social health markers

Table S1 | Sensitivity Analysis: Associations between social health markers and tract-specific white matter integrity, mutually adjusted for social health markers.

Supplemental References

Supplemental Methods | DTI and white matter tract segmentation quality control.

Tensor fits were inspected for subject-motion artifacts, geometric distortions and other artifacts by reviewing axial sections of the fractional anisotropy (FA) images by a radiologist in training. In case of interference by artifacts, tract data were set to missing. Visual inspections were performed for the 50 white matter tract segmentations per tract for which the subject-specific volume was most dissimilar from the overall mean tract volume. Segmentations were rejected and coded as missing when the segmentation did not cover the majority of mean tract anatomy, and when segmentations diverged into neighboring tracts.(1)

In total, 118 participants had a rejected inspection for ≥1 tract, and 57 participants had a failed segmentation for ≥1 tract. Tracts, but not participants were excluded from the analyses.

Figure S1 | Correlation (Pearson correlation coefficient) between social health markers.

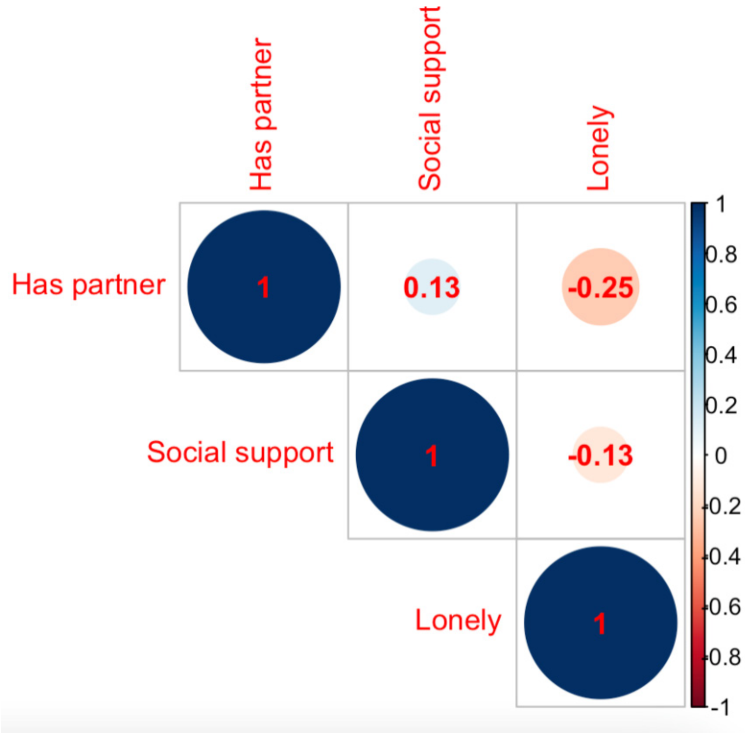

Table S1 | Sensitivity Analysis: Associations between social health markers and tract-specific white matter integrity, mutually adjusted for social health markers.

Fractional Anisotropy

		Association tracts						Commissural tracts		Limbic tracts			Sensorimotor tracts			
Social health marker	Model	ATR	IFO	ILF	PTR	SLF	UNC	FMA	FMI	CGC	CGH	FX	CST	MCP	ML	STR
Social support (per point increase)	3	0.00	0.02	0.03	0.02	0.03	0.02	0.00	0.04	0.00	0	0	0.01	-0.02	0.02	0.03
Partner status (no partner vs. partner)	3	0.01	-0.08	-0.07	-0.08	-0.02	0.00	-0.02	-0.8	-0.05	-0.03	0.08	0.01	-0.02	0.00	-0.02
Loneliness (lonely vs. not lonely)	3	-0.08	-0.05	-0.04	-0.06	-0.02	-0.07	0.00	0.00	0.07	-0.06	-0.06	0.00	0.01	0.02	0.05

Mean Diffusivity

		Association tracts						Commissural tracts		Limbic tracts			Sensorimotor tracts			
Social health marker	Model	ATR	IFO	ILF	PTR	SLF	UNC	FMA	FMI	CGC	CGH	FX	CST	MCP	ML	STR
Social support (per point increase)	3	-0.02	-0.05	-0.04	-0.01	-0.04	-0.04	0.00	-0.06	-0.03	0.00	-0.01	-0.02	0.01	-0.04	-0.01
Partner status (no partner vs. partner)	3	0.01	0.05	0.02	0.09	0.05	0.04	0.06	0.02	0.08	0.09	-0.02	0.07	0.03	0.02	0.08
Loneliness (lonely vs. not lonely)	3	0.00	0.05	0.04	0.02	0.05	0.03	0.03	0.00	0.05	0.19	-0.01	0.14	0.06	-0.02	0.20

Table S1: Standardized mean differences of model 3 including all social health variables in the same linear regression model, for fractional anisotropy (top panel) and for mean diffusivity (bottom panel). In light green the statistical significant ($p \leq 0.05$) coefficients without multiple testing correction, in dark green the statistical significant coefficients after multiple testing correction (Sidak correction). Tract abbreviations: ATR - anterior thalamic radiation, IFO - inferior fronto-occipital fasciculus, ILF - inferior longitudinal fasciculus, PTR - posterior thalamic radiation, SLF - superior longitudinal fasciculus, UNC - uncinate fasciculus, FMA - forceps major, FMI – forceps minor, CGC - cingulate gyrus part of cingulum, CGH - parahippocampal part of cingulum, FX – fornix, CST - corticospinal tract, MCP - middle cerebellar peduncle, ML - medial lemniscus, STR - superior thalamic radiation.

Supplemental References

1. de Groot M, Ikram MA, Akoudad S, Krestin GP, Hofman A, van der Lugt A, et al. (2015): Tract-specific white matter degeneration in aging: The Rotterdam Study. *Alzheimer's & Dementia*. 11:321-330.
